# Avatar Intervention for cannabis use disorder in patients with psychotic and mood disorders

**DOI:** 10.1192/j.eurpsy.2023.298

**Published:** 2023-07-19

**Authors:** S. Giguère, A. Dumais, S. Potvin

**Affiliations:** Psychiatrie, Université de Montréal, Montréal, Canada

## Abstract

**Introduction:**

Cannabis use disorder (CUD) is a complex issue, even more so when it is comorbid with a psychotic disorder or a mood disorder. Indeed, this population seems more vulnerable to this substance since the incidence of developing CUD is five to six times higher. Psychotherapies have only shown a modest short-term effect that is not maintained in the long term. The emergence of the use of virtual reality (VR) in psychiatry could be the tool that will make it possible to overcome this lack of efficiency. Indeed, VR has shown its considerable potential in a variety of psychiatric conditions. However, this modality has not been investigated for treatment for CUD. The Avatar intervention for substance use disorder allows the creation of an avatar and voice transformation that represents a significant person in relation to the patient’s substance use and is interpreted by the therapist. During the immersive sessions, patients are invited to work with their avatars on self-affirmation and refusal techniques, the management of negative emotions, stress and cravings, conflict resolution, self-esteem and motivation for change.

**Objectives:**

The purpose of this pilot project is to collect preliminary data on a new intervention.

**Methods:**

For the realization of this research project, we intend to recruit 40 participants, aged 18 and over, with a diagnosis of a CUD of at least moderate intensity, with a regular cannabis use and who also has a psychotic and/or mood disorder. The intervention consists of 8 weekly one-hour long sessions. Clinical research interviews were conducted and after therapy, and follow-ups occured at 3, 6 and 12 months. These evaluations will allow us to analyze the quantity of consumption, the severity of CUD measured with the cannabis use problem identification test (CUPIT), as well as objectively the concentration of THC thanks to samples. urinary tests which are carried out at the first and last intervention session as well as at the 3-month follow-up. Also, the psychotic symptoms and the quality of life will be evaluated.

**Results:**

In November 2022, 32 participants had been recruited of which 17 participants had completed the Avatar intervention. Preliminary results from this sample show that a decrease of moderate effect size for amount of cannabis consumed was observed, as well as on severity of the cannabis use problem after the intervention. The quantity of cannabis consumed and the severity of the problem was also significantly reduced at follow-up Quality of life tends to increase and disease symptoms decreased significantly at 3 and 6 month follow-up.

**Conclusions:**

To our knowledge, this intervention is a first in the world to target CUD in this particular population while using innovative technology The Avatar intervention under study in this project presents itself as a new avenue for cannabis use disorders.

**Disclosure of Interest:**

None Declared

